# Immunodetection of Pectic Epitopes, Arabinogalactan Proteins, and Extensins in Mucilage Cells from the Ovules of *Pilosella officinarum* Vaill. and *Taraxacum officinale* Agg. (Asteraceae)

**DOI:** 10.3390/ijms21249642

**Published:** 2020-12-17

**Authors:** Bartosz J. Płachno, Małgorzata Kapusta, Piotr Świątek, Piotr Stolarczyk, Janusz Kocki

**Affiliations:** 1 Department of Plant Cytology and Embryology, Institute of Botany, Faculty of Biology, Jagiellonian University in Kraków, 9 Gronostajowa St., 30-387 Kraków, Poland; 2 Department of Plant Cytology and Embryology, University of Gdańsk, 59. Wita Stwosza St., 80-308 Gdańsk, Poland; malgorzata.kapusta@ug.edu.pl; 3 Institute of Biology, Biotechnology and Environmental Protection, Faculty of Natural Sciences, University of Silesia in Katowice, 9 Bankowa St., 40-007 Katowice, Poland; piotr.swiatek@us.edu.pl; 4 Department of Botany, Physiology and Plant Protection, Faculty of Biotechnology and Horticulture, University of Agriculture in Kraków, 29 Listopada 54 Ave., 31-425 Kraków, Poland; stolarczykp@interia.pl; 5 Department of Clinical Genetics, Medical University of Lublin, 11 Radziwiłowska St., 20-080 Lublin, Poland; januszkocki@umlub.pl

**Keywords:** mucilage cells, ovule, dandelions, hawkweeds, Asteraceae, apomixis, arabinogalactan proteins, pectins, extensins

## Abstract

The main aim of this study was to compare the cytological difference between ovular mucilage cells in two Asteraceae species—*Pilosella officinarum* and *Taraxacum officinale*—in order to determine whether pectic epitopes, arabinogalactan proteins, or extensins are present. The immunocytochemical technique was used. Both the *Taracacum* and *Pilosella* genera have been used recently as models for understanding the mechanisms of apomixis. Knowledge of the presence of signal molecules (pectic epitopes, arabinogalactan proteins, and extensins) can help better understand the developmental processes in these plants during seed growth. The results showed that in *Pilosella officinarum*, there was an accumulation of pectins in the mucilage, including both weakly and highly esterified pectins, which was in contrast to the mucilage of *Taraxacum officinale*, which had low amounts of these pectins. However, *Taraxacum* protoplasts of mucilage cells were rich in weakly methyl-esterified pectins. While the mucilage contained arabinogalactan proteins in both of the studied species, the types of arabinogalactan proteins were different. In both of the studied species, extensins were recorded in the transmitting tissues. Arabinogalactan proteins as well as weakly and highly esterified pectins and extensins occurred in close proximity to calcium oxalate crystals in both *Taraxacum* and *Pilosella* cells.

## 1. Introduction

There are two main types of mucilage cells in plant seeds. The first type is mucilage cells that secret mucilage outside the cell to form a mucilage drop (e.g., mucilage cells of the trapping glands of carnivorous plants of genera *Drosera*, *Drosophyllum*, *Pinguicula*, and *Byblis* [1,2,3,4] or root cells [5]). The second type is mucilage cells that produce and collect mucilage inside a cell (at least during part of the cell life). Several subtypes can be distinguished in the second group. The first subtype is epidermal mucilage cells of the seeds, e.g., *Plantago* [6,7,8], members of the Brassicaceae family including the model plant species *Arabidopsis thaliana* (e.g., [9,10,11,12]), Sapindaceae [13], fruits such as Asteraceae cypsela [14,15], and Asteraceae cypsela epidermal trichomes [16]. The second subtype is epidermal and mesophyll mucilage cells of dicotyledons, in which the mucilage accumulates between the plasmalemma and the cell wall, e.g., *Opuntia ficus-indica* [17], *Cinnamomum burmanni* [18], and *Hibiscus schizopetalus* [19]. The third subtype is mucilage cells in which the mucilage accumulates between the cytoplasm and the central vacuole into a specialized cavity, e.g., *Araucaria angustifoli* [20,21]. It should be noted that mucilage can also be secreted by ducts and cavities [22].

There are two main types of mucilage: cellulose mucilage and pectin mucilage. The first represents cell walls that have a swollen matrix, which causes the cellulose fibrils to become separated from each other. In the second type, which is composed mainly of pectins and hemicelluloses, there are no cellulose fibrils [23,24,25].

As previously mentioned, mucilage can be accumulated and secreted in various ways in seed plants. Detailed studies have been performed on mucilage seed cells of the model plant *A. thaliana*. In this species, mucilage is secreted into a region of the apoplast, which is adjacent to the radial and outer tangential cell walls in the mucilage seed coat cells. In a mature cell, a donut-shaped mucilage pocket surrounds the central secondary wall, called the columella [26]. Apart from *A. thaliana* [5,27,28], there have only been a few detailed studies on the direct immunocytochemistry of mucilage cells. Most researchers have only performed a biochemical analysis of mucilage.

Huang et al. [29] analyzed mucilage cells in the seed coat of *Lepidium perfoliatum* (Brassicaceae). In this species, the mucilage contains a significant amount of acidic polysaccharides as well as xyloglucans (XG), e.g., hemicellulose and β-1,3-d-glucan. Using the anti-homogalacturonan (HG) antibodies JIM5 and JIM7, it was shown that homogalacturonan with different degrees of methyl esterification occurs in the mucilage of this species. Kreitschitz and Gorb [30] performed immunolocalization of xylan and xyloglucan in the *Neopallasia pectinata* (Asteraceae) mucilage envelope as well as immunolocalization of arabinoxylan and xyloglucan in the *Linum usitatissimum* (Linaceae) mucilage envelope. Recently, Phan et al. [8] showed changes in the occurrence of pectin-associated monosaccharides during the expansion of seed mucilage in *Plantago ovata* seeds. These authors showed that there was a different developmental pattern in *Plantago* compared to the pattern in mucilage cells of *A. thaliana*.

Mastroberti and Mariath [21] showed a gradient of distribution in relation to pectic de-esterification as well as the increase of galactan and arabinan epitope distribution during the development of mucilage cells in *Araucaria angustifolia* (Araucariaceae (gymnosperm)).

**Figure 1:**
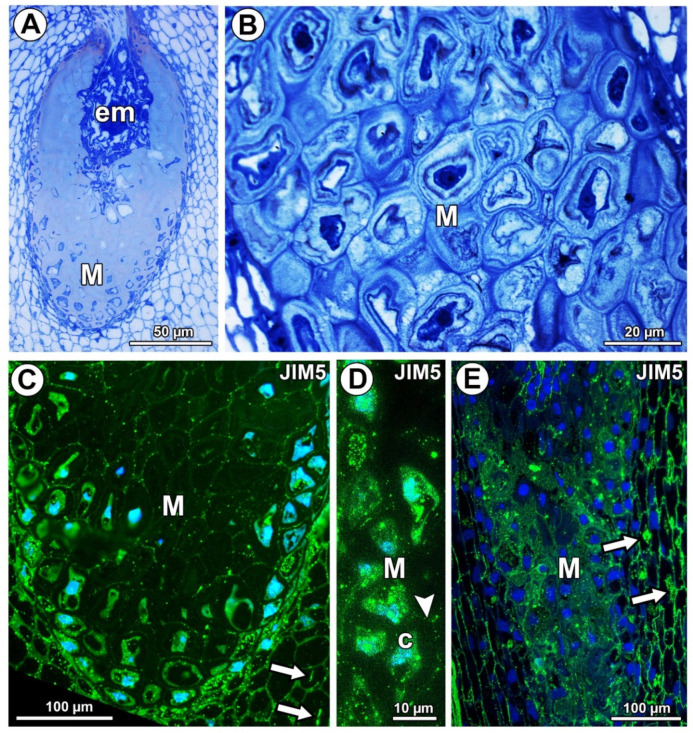
Histology and pectin (JIM5) detection. (**A**) Section through the young seed of *Taraxacum officinale*; note the presence of embryo (em) and mucilage cells (M). (**B**) Mucilage cells (M) stained with metachromatic dye toluidine blue O in an ovule of *T. officinale*. (**C**) Pectin (JIM5) detection in mucilage cells of integument periendothelial tissue in an ovule of *T. officinale*. (**D**) Pectin (JIM5) detection in mucilage cells (M); note the intense signal in the cytoplasm of mucilage cells (c) and in the cell wall (arrowhead) in an ovule of *T. officinale*. (**E**) Pectin (JIM5) detection in the ovule of *Pilosella officinarum*; mucilage cells of integument periendothelial tissue (M).

The ovule mucilage cells (periendothelial tissue) of *Pilosella* and *Hieracium* belong to the type where mucilage is accumulated between the plasmalemma and the cell wall [31]. This type of mucilage accumulation also occurs in other Asteraceae genera that have the *Taraxacum* ovule type (Figure 1A,B) [32,33]. During the maturation of *Pilosella*, *Hieracium*, and *Taraxacum*, periendothelial tissue cells undergo an intense period of secretory activity that leads to dramatic cytological changes. According to Koltunow et al. [34], there is an intensive liquefaction of these cells in *Hieracium*. Płachno et al. [31,35] observed that the mucilage first pushes the protoplast to the center of the cell. Cytoplasmic bridges are then formed and connect the protoplast to the plasmodesmata through the mucilage layers. Then, the cell walls are broken down, and lysigenous cavities filled with mucilage are formed. In a previous paper about the immunocytochemistry of Asteraceae ovules [36], arabinogalactan proteins (AGPs), hemicelluloses, and pectic epitopes in the transmitting tissue of *Taraxacum* ovules were analyzed; however, the mucilage cells were not analyzed.

The genera *Hieracium* and *Pilosella* have recently been used as models for understanding the mechanisms of apomixis [37,38,39,40]. Recently, Juranić et al. [41] used 17 monoclonal antibodies that were directed against cell wall carbohydrate epitopes to determine the presence of glycan motifs in the ovule cells of *Pilosella piloselloides* (syn. *Hieracium piloselloides*) and *Hieracium praealtum*. However, the authors mainly focused on the young ovule stages. Using the JIM13 antibody, they showed that arabinogalactans are early markers of both the sexual and apomictic cell lineages in *Hieracium* spp. Additionally, these arabinogalactans occurred in the cell walls of both sexual and aposporous female gametophytes during cellularization and maturation as well as in the micropylar cells of the ovule.

To date, there have been no detailed studies on the presence of arabinogalactans and pectins in nonepidermal mucilage cells of Asteraceae ovules. Knowledge of the presence of signal molecules (pectic epitopes, arabinogalactan proteins, or extensins) in ovules of these plants can help better understand the developmental processes during seed growth. Both the *Pilosella* and *Taraxacum* genera have been recently used as models for understanding the mechanisms of apomixis in angiosperms. Thus, the main aim of this study was to determine which arabinogalactan proteins, extensins, and pectic epitopes occur in the mucilage cells of ovules/young seeds of the apomictic species *Pilosella officinarum* and *Taraxacum officinale*. The other reason for undertaking this research was because mucilage cells in these species had previously been tested for ultrastructure and changes during mucilage production. These cells can be treated as an attractive model for research into changes in intracellular transport and mucilage structure and formation.

Recently, Leszczuk et al. [42] showed an association between arabinogalactan proteins and calcium oxalate crystals in the ovaries of *Fragaria* × *ananassa*. Calcium oxalate crystals are a common characteristic of ovular cells of Asteraceae [43,44,45]. Therefore, an additional aim was to determine whether the association between arabinogalactan proteins and calcium oxalate crystals also occurs in Asteraceae.

## 2. Results

### 2.1. Pectins

In *Taraxacum* and *Pilosella*, weakly methyl-esterified pectins, homogalacturonans labeled with JIM5, occurred in the cell walls of different ovule tissue cells (Figure 1C,E). In the mucilage cells of *Taraxacum*, these pectins occurred in the cell walls and protoplasts, but there was less intense labeling in the mucilage (Figure 1C,D). However, in *Pilosella,* these pectins especially occurred in mucilage (Figure 1E). The pectins labeled with JIM5 were also associated with calcium oxalate crystals in the parenchyma ovule cells of both taxa (Figure 1C,E).

**Figure 2:**
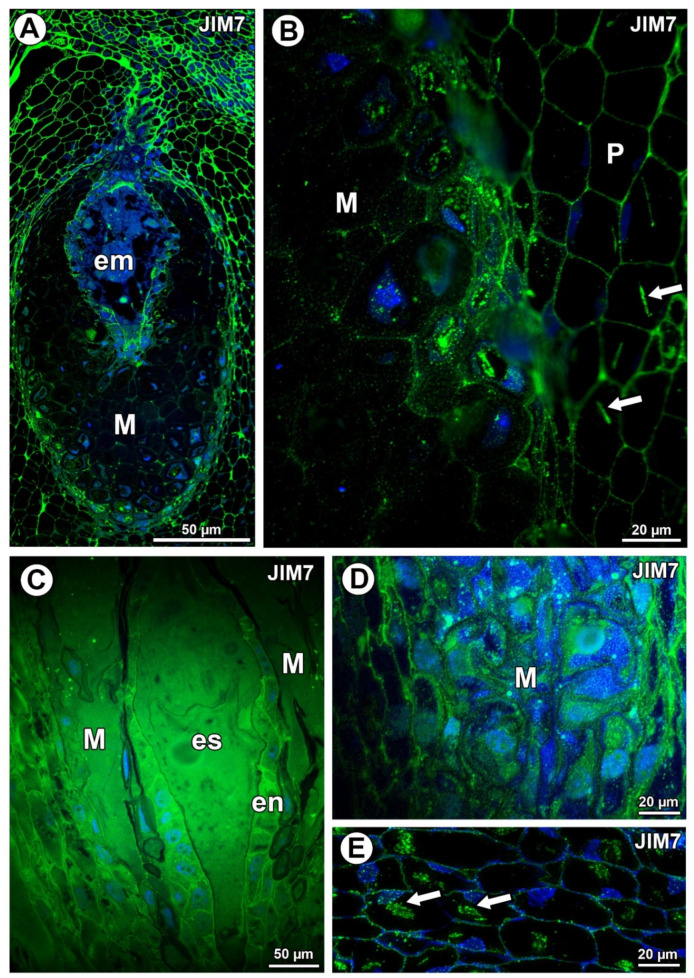
Pectin (JIM7) detection. (**A**) Distribution of the JIM7 antibody in the young seed of *Taraxacum officinale*; embryo (em) and mucilage cells of integument periendothelial tissue (M). (**B**) Pectin (JIM5) detection in mucilage cells (M) and parenchyma cells (P) in *T. officinale*; note the association between pectin and calcium oxalate crystals (arrows). (**C**) Pectin detection in ovule of *Pilosella officinarum*; embryo sac (es), mucilage (M), and endothelium (en). (**D**) Pectin detection in mucilage cells of *P. officinarum*. (**E**) Pectin association with calcium oxalate crystals (arrows) in ovule parenchyma cells of *P. officinarum*.

**Figure 3:**
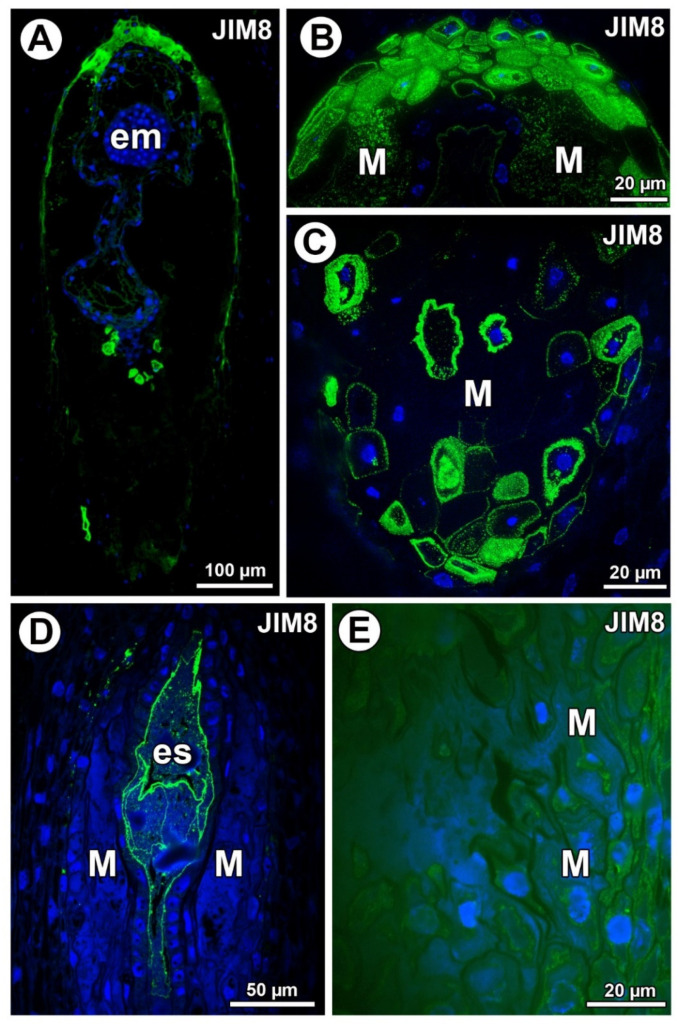
Arabinogalactan proteins (labeled with JIM8) detection. (**A**) Section through the young seed of *Taraxacum officinale*; note the presence of embryo (em). (**B**) Higher magnification of micropylar pole of *T. officinale* seed; note that arabinogalactan proteins (AGPs) occurred in the mucilage in lysigenous cavities (M). (**C**) Section through the chalazal part of young seed of *T. officinale* with mucilage cells (M). (**D**) Section through the ovule of *Pilosella officinarum*; note the presence of embryo sac (es). (**E**) Detection of AGPs in protoplasts of various ovular cells of *P. officinarum*; mucilage cells (M).

In *Taraxacum*, the highly esterified pectins, homogalacturonans labeled with JIM7, occurred in the cell walls of various ovule cell types (Figure 2A,B). Additionally, in *Pilosella,* intense labeling of the cell walls of endothelium cells occurred (Figure 2C). In the mucilage cells of *Taraxacum*, these pectins were recorded in the cell walls (intense signal) and in mucilage (much less intense signal) (Figure 2B). These pectins were associated with calcium oxalate crystals (Figure 3B). In *Pilosella* these pectins mainly occurred in the mucilage in the mucilage cells (Figure 2D). Moreover, in *Pilosella*, in the lysigenous cavities filled with mucilage, the pectins labeled with JIM7 occurred as granules (Figure 2D). The pectins labeled with JIM7 were also associated with calcium oxalate crystals in the parenchyma ovule cells of both taxa (Figure 2B,E).

### 2.2. Arabinogalactan Proteins

In *Taraxacum*, AGPs (labeled with JIM8) were detected in the mucilage cells (Figure 3A). AGPs mainly occurred in the mucilage cells at the micropylar pole (Figure 3B) and chalazal pole (Figure 3C). In *Taraxacum,* AGPs occurred in both cell walls and mucilage, which was in contrast to *Pilosella*, where an intense accumulation of AGPs was detected in the embryo sac and also in the protoplasts of the endothelium cells. In some ovules of *Pilosella*, they also occurred in the protoplasts of various ovular cells, including mucilage cells (Figure 3E); however, AGPs labeled with JIM8 were absent in the mucilage (Figure 3D,E). In the mucilage of *Taraxacum,* the AGP signal was heterogeneous. It had several distinct layers (Figure 3B) that stained differently when labeled with JIM8 (Figure 2B,C). Lack of detection of AGPs near calcium oxalate crystals was observed for both species.

**Figure 4:**
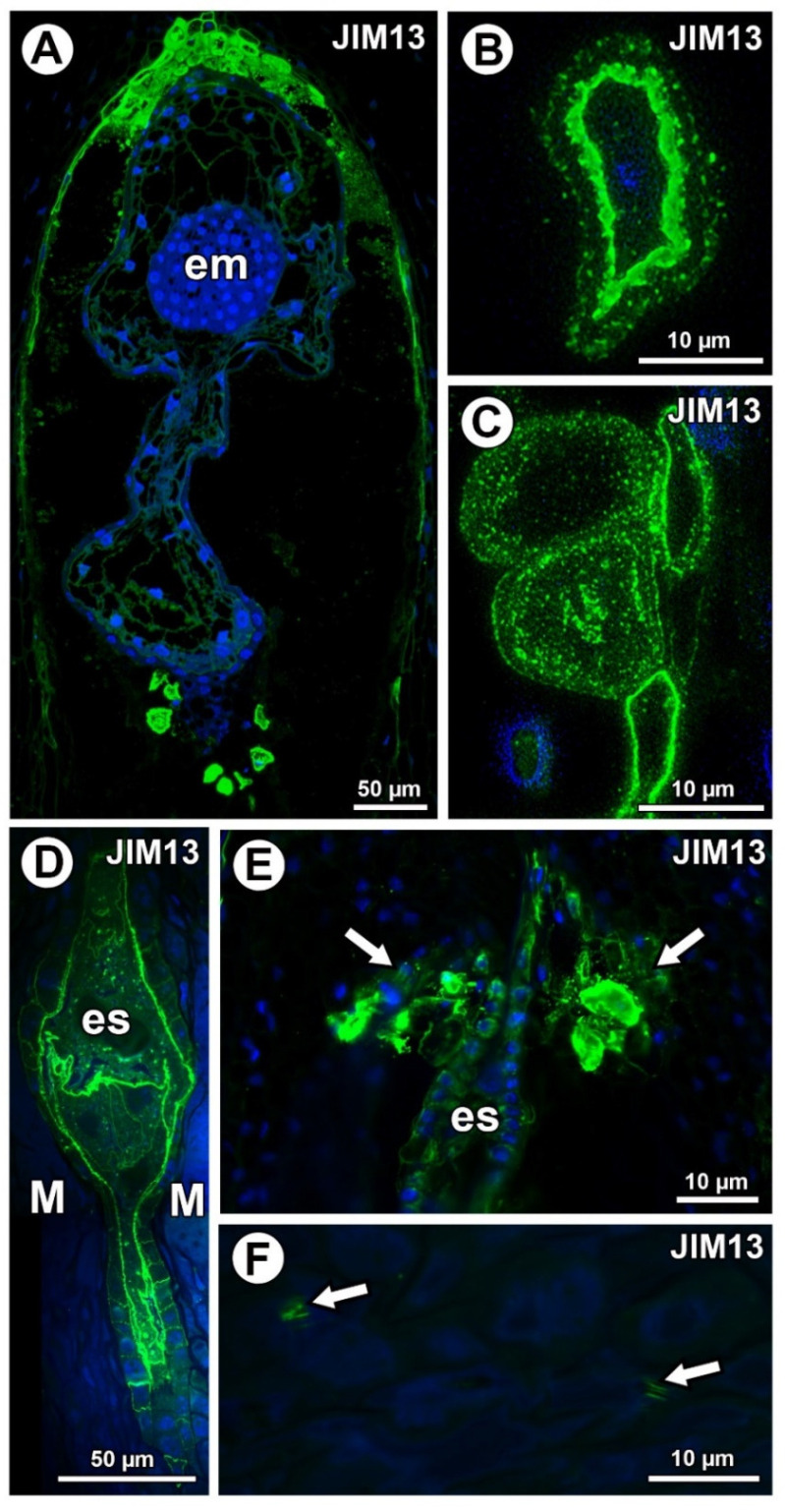
Arabinogalactan protein (labeled with JIM13) detection. (**A**) Sections through the young seed of *Taraxacum officinale*; note the presence of embryo (em). (**B**,**C**) AGP detection in mucilage cells in the young seed of *T. officinale*; note several distinct layers in the mucilage. (**D**,**E**) Occurrence of AGPs in embryo sac (es), endothelium, and in group of mucilage cells at micropylar pole (arrows) in *Pilosella officinarum* ovules. (**F**) Association between AGPs and calcium oxalate crystals (arrows) in ovule parenchyma cells of *P. officinarum*.

In *Taraxacum*, AGPs labeled with JIM13 were present (Figure 4A,C), which was similar to AGPs labeled with JIM8 in this species. Moreover, AGPs were stained differently in the layers of the mucilage (Figure 4B,C). In *Pilosella*, AGPs labeled with JIM13 were detected in cell walls in the parenchyma ovule cells and some mucilage cells, primarily those near the embryo sac at the micropylar pole (Figure 4D,E). Although there were no AGPs in chalazal mucilage cells, they were associated with calcium oxalate crystals in the parenchyma ovule cells (Figure 4F).

**Figure 5:**
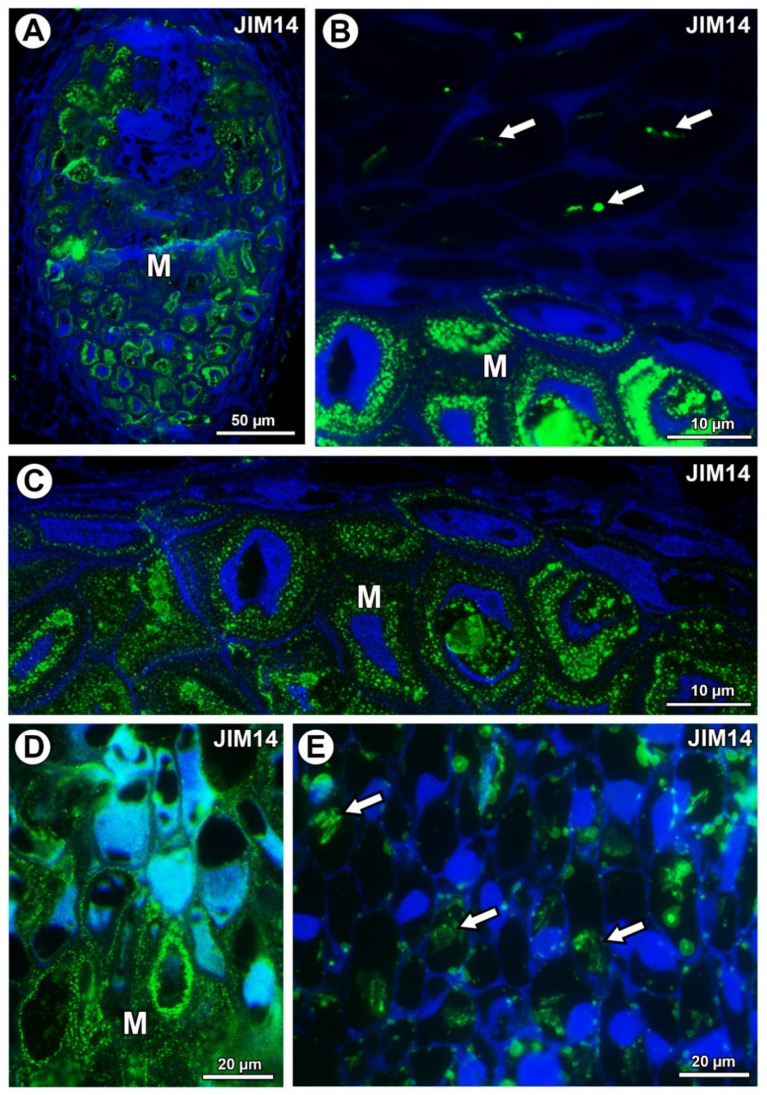
Arabinogalactan protein (labeled with JIM14) detection. (**A**) Section through the young seed of *Taraxacum officinale*; mucilage cells (M). (**B**) Association between AGPs and calcium oxalate crystals (arrows) in parenchyma cells; mucilage cells (M) in the young seed of *T. officinale*. (**C**) AGP detection in mucilage cells (M); note several distinct layers in the mucilage in the young seed of *T. officinale.* (**D**) AGP detection in mucilage cells (M) in *Pilosella officinarum* ovule. (**E**) Association between AGPs and calcium oxalate crystals (arrows) in ovule parenchyma cells of *P. officinarum* ovule.

For both examined species, AGPs labeled with JIM14 occurred in the mucilage of the mucilage cells (Figure 5A,D). These AGPs were also associated with calcium oxalate crystals in the parenchyma ovule cells of *Taraxacum* (Figure 5B) and likewise for *Pilosella* (Figure 5E).

**Figure 6:**
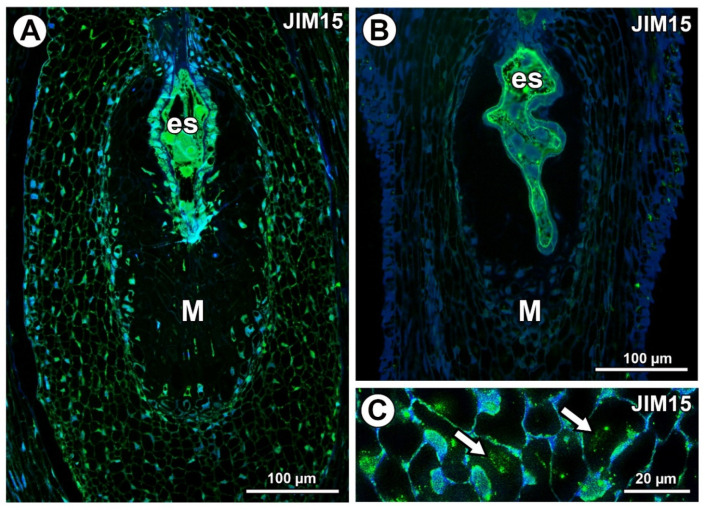
Arabinogalactan protein (labeled with JIM15) detection. (**A**) Section through the young *Taraxacum* seed; note the presence of embryo sac (es) and mucilage cells (M). (**B**) Section through the *Pilosella officinarum* ovule, with AGPs occurring in embryo sac; note the presence of mucilage cells (M). (**C**) Association between AGPs and calcium oxalate crystals (arrows) in ovule parenchyma cells of *P. officinarum*.

AGPs labeled with JIM15 were only observed in some protoplasts of some mucilage cells of the *Taraxacum* ovules; however, they were absent in the mucilage (Figure 6A). In *Pilosella*, AGPs labeled with JIM15 occurred in the embryo sac, endothelium cells, and transmitting tissue, but they were absent in the mucilage cells (Figure 6B). These AGPs were associated with calcium oxalate crystals in the parenchyma ovule cells (Figure 6C).

**Figure 7:**
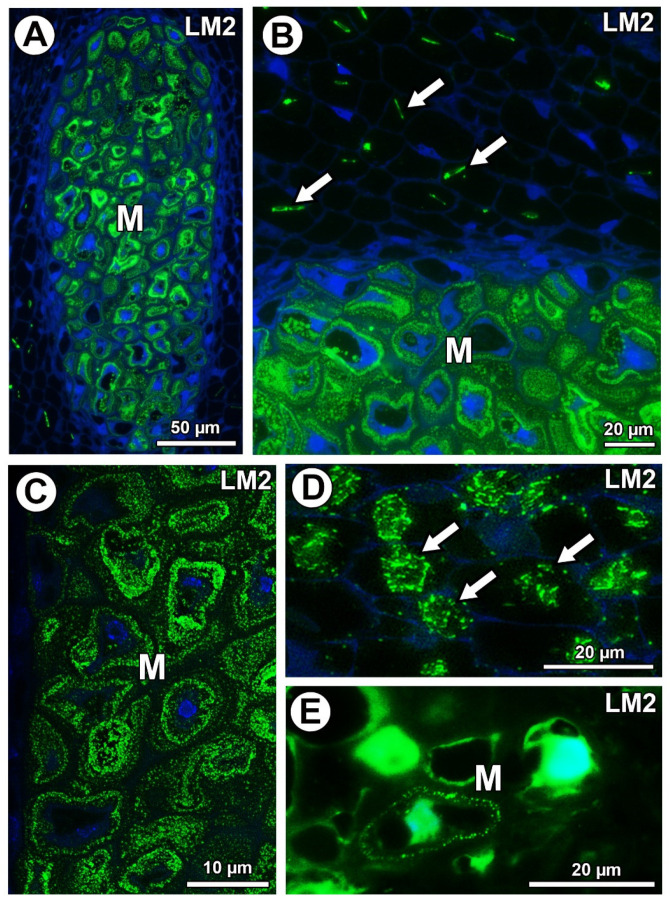
Detection of arabinogalactan proteins (labeled with LM2). (**A**) Section through the young seed of *Taraxacum officinale*; AGPs occurred in mucilage cells (M). (**B**) AGP detection in mucilage cells (M) and association between AGPs and calcium oxalate crystals (arrows) in parenchyma cells in the young seed of *T. officinale*. (**C**) AGP detection in mucilage cells (M); note several distinct layers in the mucilage in the young seed of *T. officinale*. (**D**) Association between AGPs and calcium oxalate crystals (arrows) in parenchyma cells of *Pilosella officinarum* ovule. (**E**) AGP detection in mucilage cells (M) of *P. officinarum* ovule.

In *Taraxacum* and *Pilosella*, AGPs labeled with LM2 occurred in the mucilage of the mucilage cells (Figure 7A–C,E). Moreover for *Taraxacum*, AGPs were stained differently in layers of the mucilage (Figure 7A–C), while for *Pilosella*, these AGPs only occurred in a few mucilage cells where they formed a layer between the mucilage and the protoplast (Figure 7E). For both species, these AGPs were also associated with calcium oxalate crystals in the parenchyma ovule/seed cells (Figure 7B,D).

**Figure 8:**
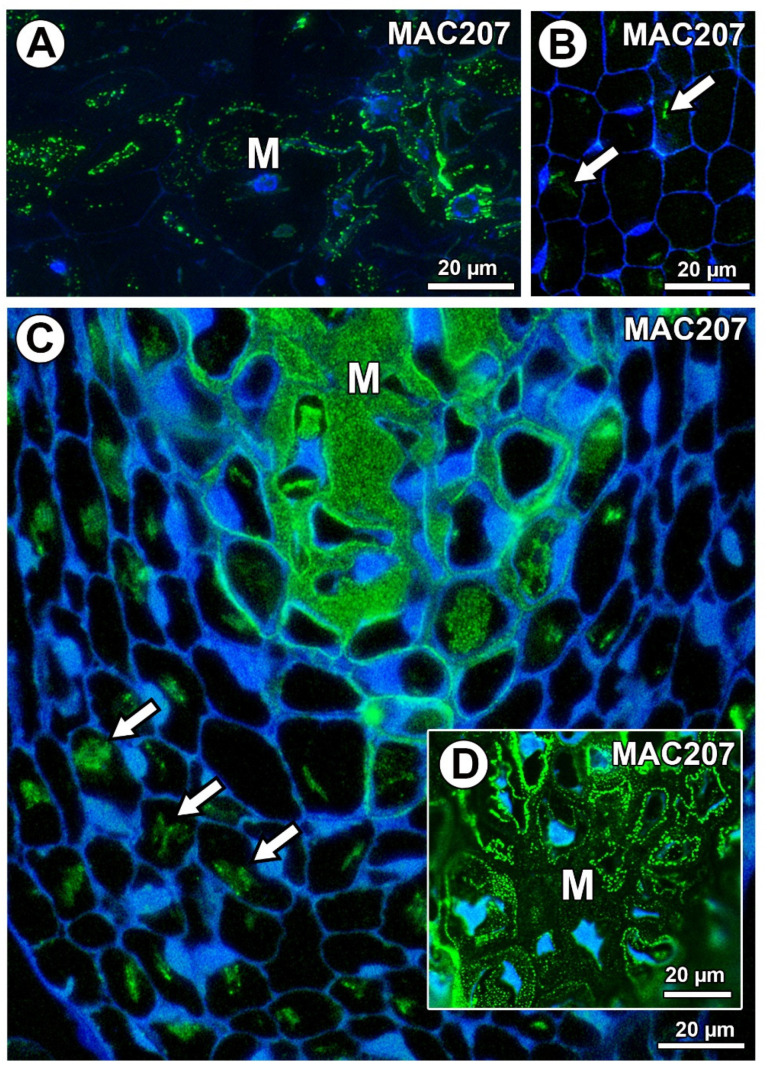
Arabinogalactan protein (labeled with MAC207) detection. (**A**) AGP detection in mucilage cells (M) in *Taraxacum officinale* ovule. (**B**) Association between AGPs and calcium oxalate crystals (arrows) in parenchyma cells of *T. officinale* ovule. (**C**,**D**) Section through the ovule of *Pilosella officinarum*; note the occurrence of AGPs in the mucilage cells (M) and the association between AGPs and calcium oxalate crystals (arrows).

In *Taraxacum* and *Pilosella*, AGPs labeled with MAC207 occurred in the mucilage cells (Figure 8A,C,D), especially in the cytoplasm (Figure 8A). However, in some mucilage cells of *Pilosella*, there was an especially intense labeling between the mucilage and the protoplasts (Figure 8D). For both species, these AGPs were also associated with calcium oxalate crystals in the parenchyma ovule cells (Figure 8B,C).

### 2.3. Extensins

**Figure 9:**
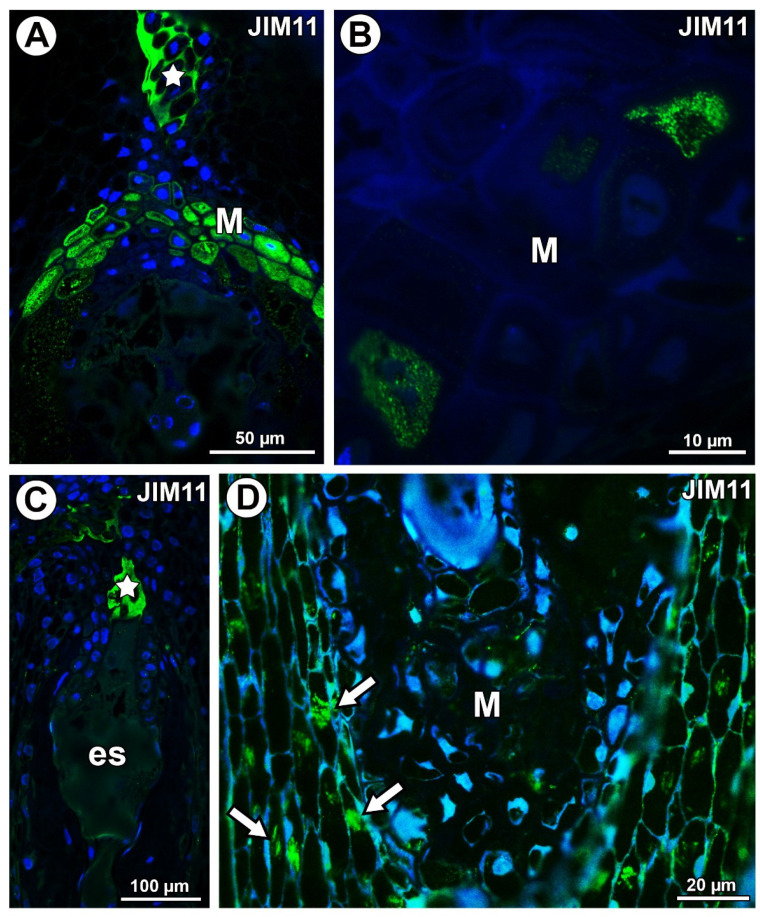
Extensin (labeled with JIM11) detection. (**A**) Section through the young seed of *Taraxacum officinale*; note the occurrence of extensins in the transmitting tissue (star) and mucilage cells in the micropylar pole (M). (**B**) Extensin detection in cytoplasm of some mucilage cells (M) of *T. officinale* young seed. (**C**) Section through the ovule of *Pilosella officinarum*; note the occurrence of extensins in the transmitting tissue (star); embryo sac (es). (**D**) Association between extensions and calcium oxalate crystals (arrows); note the mucilage cells (M) in *P. officinarum* ovule.

In *Taraxacum*, the extensins labeled with JIM11 occurred in the transmitting tissue and in a group of mucilage cells at the micropyle pole in the border between the endosperm and the integument (Figure 9A). Extensins were also recorded in a few mucilage cells in the cytoplasm of these cells (Figure 9B). Meanwhile, in *Pilosella*, extensins labeled with JIM11 occurred only in the transmitting tissue (Figure 9C). These extensins in *Pilosella* were also associated with calcium oxalate crystals (Figure 9D). However, they were not recorded in the mucilage cells (Figure 9D).

**Figure 10:**
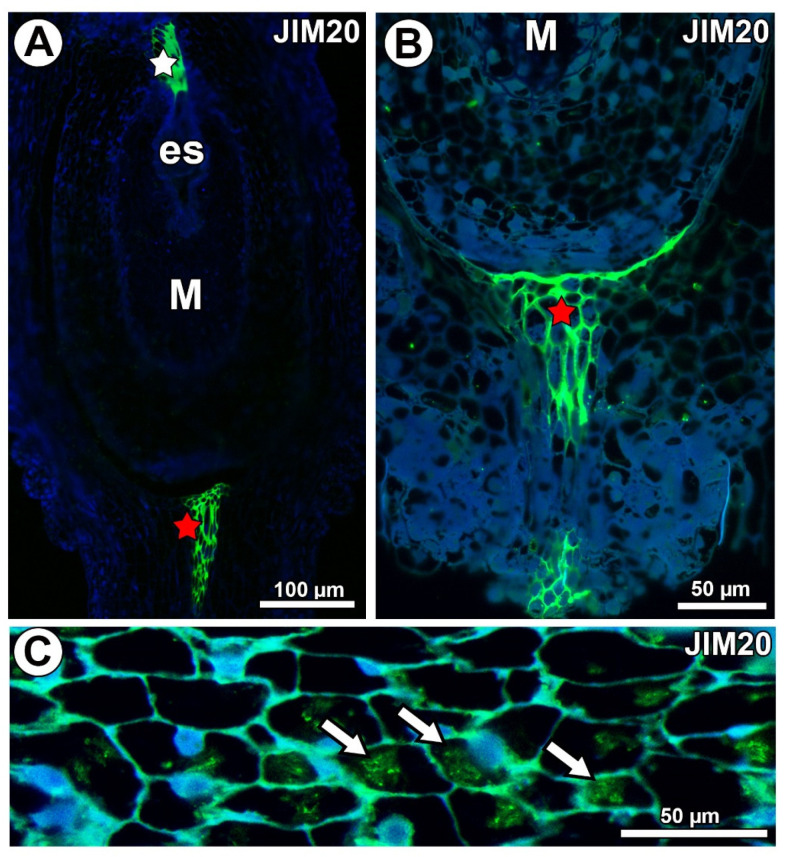
Extensin (labeled with JIM20) detection. (**A**) Section through the ovule of *Taraxacum officinale*; note the mucilage cells (M) and occurrence of extensins in the transmitting tissue (white star) and pistil transmitting tissue (red star). (**B**) Occurrence of extensins in pistil transmitting tissue of *Pilosella officinarum*. (**C**) Association between extensins and calcium oxalate crystals (arrows) in *P. officinarum* ovule.

In *Taraxacum* and *Pilosella*, extensins labeled with JIM20 occurred in the ovule transmitting tissue (Figure 10A) and in the pistil transmitting tissue (Figure 10A,B). Extensins labeled with JIM20 were only associated with calcium oxalate crystals in *Pilosella* (Figure 10C). They were not recorded in the mucilage cells (Figure 10A,B) for both species.

## 3. Discussion

### 3.1. Pectins

The pectinaceous nature of *Taraxacum* and *Pilosella* mucilage is evident from staining with periodic acid–Schiff (PAS) [31,46], ruthenium red [31], and metachromatic dye toluidine blue O (Figure 2B). In both *Taraxacum* and *Pilosella*, esterified pectins were recorded in the mucilage cells, e.g., in the cell walls. However, there were differences. The *Pilosella* mucilage contained both weakly and highly methyl-esterified pectins (intensive labeling with JIM5 and JIM7, respectively), which was in contrast to the mucilage of *Taraxacum*. However, in *Taraxacum*, protoplasts of mucilage cells had weakly methyl-esterified pectins that occurred as granules (intensive labeling). Moreover, the seed mucilage of *A. thaliana* had a pectinaceous nature, with both weakly and highly methyl-esterified pectins recoded [5,27]. Macquet et al. [27] showed that pectins labeled with JIM5 were not evenly distributed, the labeling was punctate, and there were regions where the labeling was denser. Pectins labeled with JIM7 occurred at the outer edge of the inner mucilage layer. Thus, in the *A. thaliana* mucilage (in the inner mucilage layer), the methyl-esterified pectins formed two distinct populations [27]. According to Huang et al. [29], one major component of the seed coat mucilage in *Lepidium perfoliatum* could be the highly methyl-esterified pectins. These authors observed only a weak labeling with JIM5 in the mucilage. The mucilage from the immature and mature leaves of *Araucaria angustifolia* was very low in the weakly methyl-esterified pectins, and it was low in the highly methyl-esterified pectins, which was in contrast to the cell walls of mucilage cells [21].

Methyl-esterified pectins are important as signal molecules, but the degree of their esterification affects the properties of both the cell walls and the mucilage. This may result in a variation in occurrence of different populations of methyl-esterified pectin in mucilage cells, not only in *Taraxacum* and *Pilosella* but also in other plants.

### 3.2. AGPs

Most authors have regarded AGPs as embryo sac markers in flowering plants that have a sexual mode of reproduction. AGPs have been found in the cell wall of the central cell and in cells of the egg apparatus [47,48,49,50,51,52]. JIM8 and JIM13 showed an especially strong and specific labeling in the embryo sac wall and egg apparatus as well as in the pathway of pollen tube growth [47,48,51]. Additionally, AGPs play an important role in sexual reproduction in conifers [53]. The situation is more complicated in apomictic plants. For example, in *Chondrilla juncea* (an obligate apomictic with the diplosporous embryo sac of *Taraxacum* type), AGPs labeled with JIM8 were not detectable at any of the developmental stages of the ovules [54]. Moreover, these authors observed the absence of AGPs labeled with JIM13 inside the micropylar canal and mature embryo sac of this species. In contrast to *Chondrilla*, Gawecki et al. [36] observed the occurrence of AGPs in the cytoplasm of synergids and in cytoplasm and the cell walls of the central cell in apomictic *Taraxacum*. These authors also observed AGPs in the pathway of pollen tube growth in *Taraxacum* ovules. However, similar to the results obtained with *Chondrilla* [54], AGPs labeled with JIM8 were not detected in the ovules of *Taraxacum* [36]. However, in this study, we observed these AGPs in *Taraxacum* ovules. Recently, Rojek et al. [55] found AGPs labeled with JIM13 in the initial cell, which is destined to form the functional megaspore in apomictic *Boechera stricta*. We observed AGPs labeled with JIM13, JIM8, and JIM15 in the cell walls of embryo sacs in apomictic *Pilosella piloselloides*. Thus, our results are in agreement with Juranić et al. [41], who also observed AGPs labeled with JIM13 in the cell walls of embryo sacs in apomictic *Pilosella*. Thus, AGPs occur in the female gametophytes in both amphimictic and apomictic taxa. However, it should be noted that according to some authors, *Pilosella piloselloides* hexaploids are either sexual or apomictic [52,56]. Therefore, the *Pilosella piloselloides* complex requires further cytoembryological investigations.

Typically, AGPs are anchored to the extracellular side of the plasma membrane, thereby forming a narrow AGP-rich region between the cell membrane and the cell wall proper [57,58,59,60]. However, in mucilage cells of both *Taraxacum* and *Pilosella*, the periplasmic space was broad and increased during maturation of these cells. While we found that the *Taraxacum* mucilage was reached in various AGPs (labeled with JIM8, JIM13, JIM14, and LM2), there were clear similarities and differences between AGPs in them. The labeling with JIM8 and JIM13 was very similar, namely the mucilage cell group at the micropylar pole and some mucilage cells at the chalazal pole. Only some of the mucilage cells were labeled. In contrast to this, AGPs labeled with JIM14 and LM2 were present in all of the mucilage cells. Gawecki et al. [36] also observed the accumulation of AGPs labeled with JIM13 in mucilage cells near the egg apparatus in *Taraxacum*. This accumulation might be connected with pollination and the growth of pollen tubes. However, we observed this accumulation at the stage when the embryo was formed. Thus, in apomictic *Taraxacum*, this accumulation of AGPs is the remains of sexual reproduction [32,61] or has a different function.

According to Leszczuk et al. [42], AGPs are involved in establishment of the cell wall, namely the plasma membrane continuum. During maturation in *Pilosella* and *Taraxacum*, mucilage cells undergo an intense period of secretory activity, which leads to dramatic cytological changes in the relationship between the cell wall and the plasma membrane [31,35].

The novelty shown here points toward a layered occurrence of AGPs in the mucilage. Earlier, Płachno et al. [35] showed the presence of stretches in the mucilage of *Taraxacum* mucilage cells using aqueous methylene blue with azure II. The presence of well visible stretches in the mucilage has also been recorded in other plants [2,62,63]. It would be quite interesting to determine whether the occurrence of these stretches is connected with the presence of AGPs.

AGPs also occurred in the *Pilosella* mucilage labeled with MAC207 and JIM14. These AGPs were rich in the mucilage layer near the plasmalemma.

Mastroberti and Mariath [21] found AGPs labeled with JIM13 in the mucilage in mucilage cells of *Araucaria angustifolia*. They showed that there was an increase in AGPs in the mucilage during the secretion stage of cells but a dramatic decrease in mature cells. These authors connected the changes in the occurrence of AGPs with the programmed cell death (PCD) process in these mucilage cells. Thus, we also suggest that the variation in the occurrence of AGPs in *Taraxacum* mucilage may be connected to the cytological events in these cells that lead to PCD.

An accumulation of AGPs in mucilage cells of the studied species indicates the increased activity of these cells and suggests that they may play an important role in communication between maternal tissues and the embryo.

### 3.3. Extensins

According to Tan et al. [64], the wall ion-regulated intermolecular interactions between AGPs and/or extensins may be involved in maintaining the integrity of the wall plasma membrane during wall loosening processes, e.g., during wall elongation or expansion. This may be a good explanation for the presence of extensins in the ovular transmitting tissue of Asteraceae. The cells of this tissue have a well-developed extracellular matrix that forms a track for pollen tube growth [32]. We observed extensins in the micropylar group of mucilage cells, and there was also an accumulation of AGPs in these cells. However, extensins can perform various roles in plants. They are involved in the plant response to stress factors [65,66,67,68], plant regeneration in in vitro conditions [69], and embryogenesis [70].

This is the first study showing the occurrence of extensins in ovules of apomictic plants. Coexistence of AGPs and extensins does not appear to be incidental and requires further investigation.

### 3.4. Calcium Oxalate Crystals

Leszczuk et al. [42] showed the presence of AGPs labeled with JIM13 and JIM15 in close proximity to calcium oxalate (CaOx) crystals in the ovary wall of *Fragaria* × *ananassa* (Rosaceae). These authors were successful in performing an immunogold reaction, which confirmed the presence of AGPs at the edge of the CaOx crystals. Here, we showed the presence of AGPs in close proximity to CaOx in Asteraceae. AGPs labeled with JIM13, LM2, and JIM14 occurred in close proximity to calcium oxalate crystals in the ovule cells of *Taraxacum*, while in *Pilosella*, there was a similar connection with AGPs labeled with JIM13, MC207, and LM2. AGPs bind and release cell-surface apoplastic calcium (AGP-Ca^2+^ capacitor calcium) [59,60,71,72]. This would explain the presence of AGPs at the edge of the CaOx crystals. Leszczuk et al. [42] proposed an attractive hypothesis: “Crystals associated with AGPs may serve dual functions: deposition of calcium ions important for the developing embryo and seed coat formation as well as involvement in the earliest events during sexual plant reproduction—the interaction between pollen tubes and female gametophyte getting ready for fertilization”. Our results are in agreement with the results of Leszczuk et al. [42]. However, we also observed an association between AGPs and CaOx in the ovules of apomicts, which do not require fertilization (*Taraxacum officinale*). Thus, in the studied apomicts, the deposition of calcium ions is probably more important for the developing embryo sac and later the embryo, endosperm, and seed coat formation than for pollen tube attraction and growth (Table 1).

The novelty observed in this work was the occurrence of weakly and highly esterified pectins and extensins in close proximity to CaOx in both *Taraxacum* and *Pilosella* cells (Table 1).

## 4. Materials and Methods

*Pilosella officinarum* Vaill. = *Hieracium pilosella* L. (hexaploid clone x = 9, [73]) plants were collected from Mt. Treskovac, Banat, Romania, and later cultivated in the garden of Bartosz Jan Płachno. Studies were conducted on the flowers both before and during anthesis. Plants that were harvested before anthesis contained a mature embryo sac, whereas plants that were harvested during anthesis already contained an embryo and endosperm, as was previously observed [31]. *Taraxacum officinale* agg. plants were collected in Gdańsk and Kraków-Podgórze, Poland. Studies were conducted on the flowers before and during anthesis.

### Sample Preparation

Information on the antibodies used in the study are presented in Table 2.

The detailed procedure for observing the histological sections and performing immunochemical analysis are given in Płachno et al. [68]. The plant material was fixed overnight at 4 °C in 8% (*w*/*v*) paraformaldehyde (PFA, Sigma-Aldrich, Sigma-Aldrich Sp. z o.o. Poznan, Poland) with 0.25% (*v*/*v*) glutaraldehyde (GA, Sigma-Aldrich, Sigma-Aldrich Sp. z o.o. Poznan, Poland) in a piperazine-N,N′-bis(2-ethanesulfonic acid (PIPES, Sigma-Aldrich, Sigma-Aldrich Sp. z o.o. Poznan, Poland) buffer. It was then embedded in LR White resin (Polysciences Europe GmbH, Hirschberg an der Bergstrasse, Germany) and sectioned into 1 µm sections, which were blocked with 1% bovine serum albumin (BSA, Sigma-Aldrich, Sigma-Aldrich Sp. z o.o. Poznan, Poland) in a phosphate-buffered saline (PBS) buffer and incubated with the primary antibodies against pectins (JIM5 and JM7), arabinogalactans (JIM8, JIM13, JIM14, JIM15, LM2, and MAC207), and extensins (JIM11 and JIM20) overnight at 4 °C. All of the primary antibodies were obtained from Plant Probes, UK, and the secondary antibody goat anti-rat conjugated with fluorescein isothiocyanate (FITC) was from Abcam (Abcam plc, registered in England and Wales with Company Number 03509322, Discovery Drive, Cambridge Biomedical Campus, Cambridge, CB2 0AX, UK). The chromatin in the nuclei was stained with 7 µg/mL 4′,6-diamidine-2′-phenylindole dihydrochloride (DAPI, Sigma-Aldrich, Sigma-Aldrich Sp. z o.o. Poznan, Poland), and the samples were then cover-slipped using a Mowiol medium. They were viewed with a Leica DM6000 B (Leica Microsystems GmbH, Wetzlar Germany) microscope using a FITC and DAPI filter. Some of the photos were acquired as *Z* stacks and deconvolved using two iterations of a 3D nonblind algorithm (Autoquant™) in order to maximize the spatial resolution, and the images are the maximum projections. At least two different replications were performed for each species and developmental stage of the analyzed flowers, and about 5–10 sections were analyzed from each organ for each antibody that was used. Negative controls were performed by omitting the primary antibody step, which resulted in no fluorescence signal in any of the control frames for any of the stained slides (Supplementary Materials, Figure S1).

## 5. Conclusions

Mucilage ovular cells of *Taraxacum* and *Pilosella* are a specific cell type that have a huge periplasmic space. This region between the cell membrane and the cell wall proper was found to be rich in AGPs. However, there were differences in the occurrence of the types of AGPs between the examined species. A layered occurrence of AGPs was observed in the mucilage. This is the first time (at least to the authors’ knowledge) that this specific arrangement of AGPs in mucilage has been presented. An accumulation of AGPs in mucilage cells of the studied species may indicate that they play an important role in communication between maternal tissues and the embryo.

In *Pilosella officinarum*, there was an accumulation of pectins, including both weakly and highly esterified pectins in the mucilage, which was in contrast to the mucilage of *Taraxacum officinale*, which was low in these pectins. In both species, extensins occurred in the transmitting tissue that form the track for pollen tube growth. This is the first time that the presence of extensins in the ovules of apomicts has been shown.

## Figures and Tables

**Table 1:** Association of calcium oxalate crystals in the parenchyma cells in *Taraxacum* and *Pilosella*.

Antibody/Species	*Taraxacum*	*Pilosella*
JIM5	+	+
JIM7	+	+
JIM8	−	−
JIM13	−	+
JIM14	+	+
JIM15	−	+
LM2	+	+
MAC207	−	+
JIM11	−	+
JIM20	−	+

**Table 2:** List of the monoclonal antibodies used in the current study, the epitopes they recognize, and references.

Antibody	Epitope	References
Pectins		
JIM5	Homogalacturonan (HG) domain of c pectic polysaccharides, recognizes partially methyl-esterified epitopes of HG and can also bind to unesterified HG	[74,75]
JIM7	HG domain of the pectic polysaccharides, recognizes partially methyl-esterified epitopes of HG but does not bind to unesterified HG	[75]
AGPs		
JIM8	Arabinogalactan	[76]
JIM13	Arabinogalactan/arabinogalactan protein	[75,77]
JIM14	Arabinogalactan/arabinogalactan protein	[75,77]
JIM15	Arabinogalactan/arabinogalactan protein	[75,77]
LM2	Arabinogalactan/arabinogalactan protein	[76,78]
MAC207	Arabinogalactan/arabinogalactan protein	[77,79]
Extensins		
JIM11	Hydroxyproline-rich glycoprotein (HRGP), periodate sensitive epitope	[80,81]
JIM20	Hydroxyproline-rich glycoprotein (HRGP), periodate sensitive epitope	[80,81]

## Supplementary Materials

Supplementary materials can be found at https://www.mdpi.com/1422-0067/21/24/9642/s1. Figure S1. Control reactions of immunolabeling of mucilage and cell wall components.
