# Supplementary material for: Immunodetection of Pectic Epitopes, Arabinogalactan Proteins, and Extensins in Mucilage Cells from the Ovules of Pilosella officinarum Vaill. and Taraxacum officinale Agg. (Asteraceae)
Source: Int J Mol Sci. 2020 Dec 17;21(24):9642. doi: 10.3390/ijms21249642 (PMC7766254; doi:10.3390/ijms21249642)
Supplement: Supplementary file 1 [file ijms-21-09642-s001.pdf]

International Journal of
Molecular Sciences

Supplementary Materials

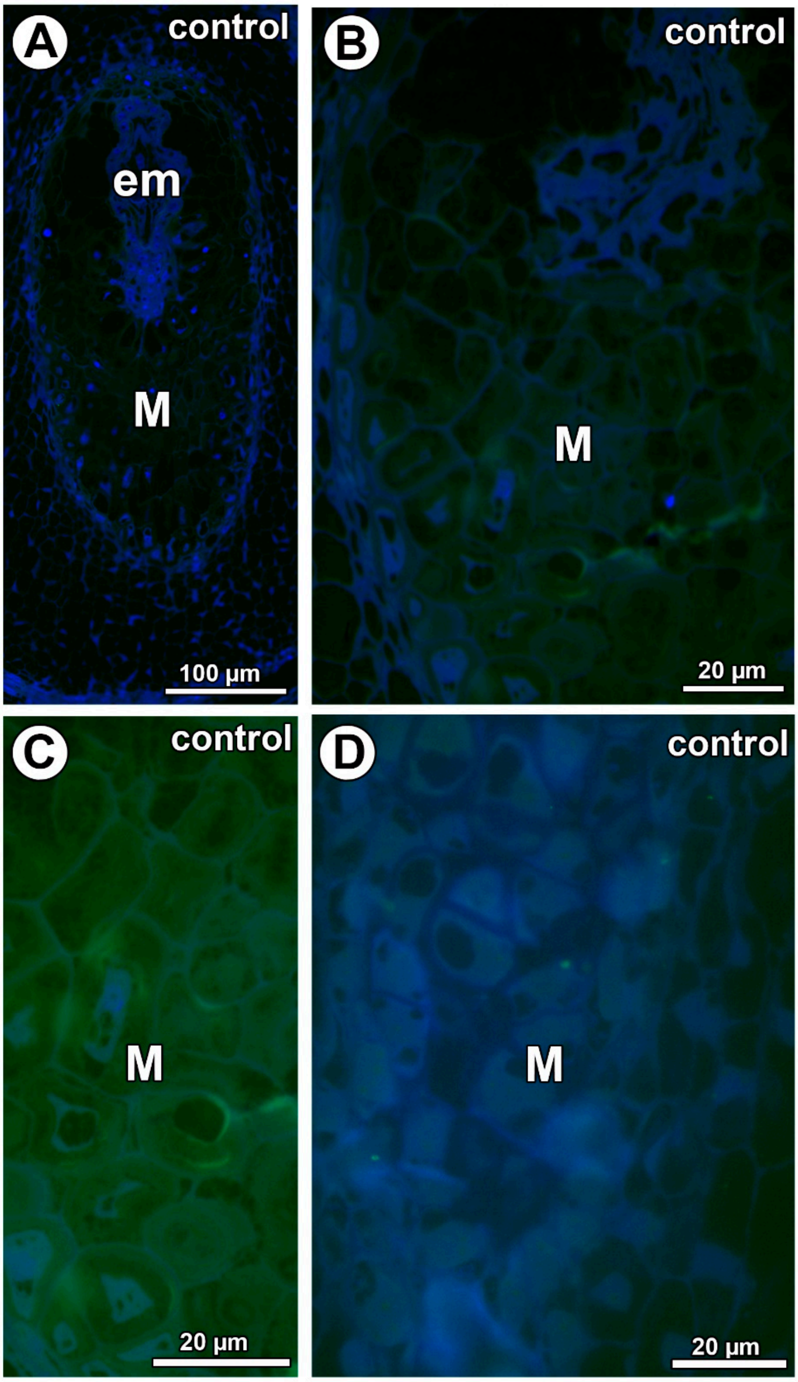

Supplementary Figure 1. Control reactions of immunolabeling of mucilage and cell wall components. (**A,B**) Section through the young seed of *Taraxacum officinale*, note embryo (em) and mucilage cells (M). (**C**) Mucilage cells (M) from *Taraxacum* seed. (**D**) Section through the ovule of *Pilosella officinarum* with mucilage cells (M).
